# Keypoints to Successful Newborn Hearing Screening. Thirty Years of Experience and Innovations

**DOI:** 10.3390/healthcare9111436

**Published:** 2021-10-25

**Authors:** Jose Miguel Sequi-Canet, Juan Brines-Solanes

**Affiliations:** 1 Department of Pediatrics, Hospital Francesc de Borja, 46701 Gandia, Spain; 2 Department of Pediatrics, University of Valencia, 46010 Valencia, Spain; juan.brines@uv.es

**Keywords:** neonatal hearing screening, otoacoustic emissions, deafness, newborn screening

## Abstract

Congenital deafness is a major pediatric problem, affecting about 1.5–3 per 1000 newborns. The early treatment through cochlear implantation and auditory rehabilitation has been a historic milestone. Early diagnosis of congenital deafness is an essential requirement to obtain the best results, which is achieved through neonatal screening, a diagnostic practice that we began systematically at the Hospital Clínico in Valencia (Spain) 30 years ago. Neonatal hearing screening is successful in most developed countries. Its implementation has been slow due to the multiple difficulties that its universal application entails since it involves several health professionals and must be carried out, in a short time interval after birth. In addition, it must have a good performance that prevents the overload of other services and that requires experience and continuous adjustments in search of proper protocols. The aim of this review is to shed some light on some key points of neonatal hearing screening, highlighting our experience in the solutions to common problems. We will discuss about techniques, protocols and neonatal or nutritional factors that can influence the screening results. To a summary of our work, an update on the subject is provided with the intention of sharing experiences and facilitating the start-up of the new units.

## 1. Introduction

**Figure 1:**
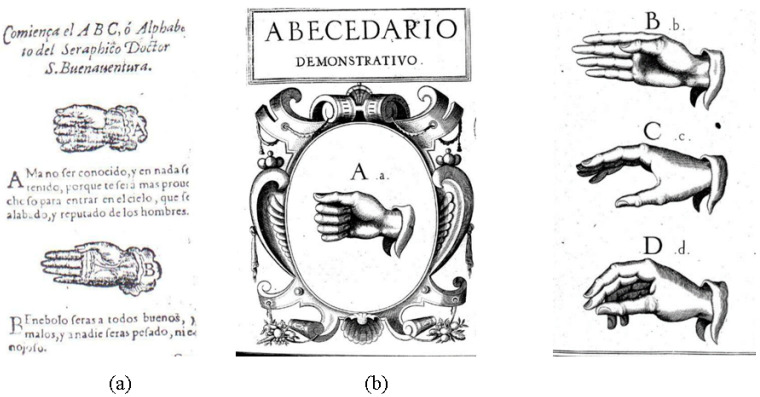
First letters of sign alphabets according to M. de Yebra (1593) [2,3] y J.P. Bonet (1620) [4].

Congenital deafness is a major pediatric problem, affecting about 1–3 per 1000 newborns [1]. Without adequate treatment, its most feared consequence is deaf-muteness. From the mid-Renaissance [2,3,4] to the end of the 20th century, most deaf children achieved social communication through sign language (Figure 1). 

The early treatment of these children through auditory rehabilitation programs for learning sound processing and cochlear implantation has been a historic milestone. This fact supports the need for the diagnosis of congenital deafness to be universal and, as soon as possible, that is, through neonatal screening. In this line, it is worth remembering that more than 90% of deaf newborns have healthy parents and that many of profoundly deaf require a cochlear implant [5,6].

Neonatal hearing screening has been successfully implemented in most Western countries. Its set-up has been slow due to the multiple difficulties that its universal application entails. The organization at a regional level is complex and the technical equipment, preparation of health personnel and administration in general makes it a hard and costly enterprise to run. It involves several care services and levels of health staff; it should be universal, and it should be carried out mainly in the short postpartum stay in maternity ward. A good performance is needed to avoid the overload of hospital services concerned. There is currently a strong discussion about the more appropriate technical equipment and protocols to be used, but all the programs manage to increase the early detection of congenital deafness in the neonatal period, which has made it possible to reduce the age of treatment and have better clinical prognosis and psychosocial adaptation [7,8,9,10].

We have systematically applied this practice at the Hospital Clínico in Valencia (Spain) during the last 30 years [11,12,13].

This review summarizes our experience over more than three decades and we will discuss the key points to achieve a satisfactory neonatal hearing screening, highlighting the solutions to the problems that usually appear in regard to equipment, techniques, protocols and neonatal or nutritional factors that can influence the results.

## 2. Neonatal Hearing Screening

Much time and effort has been devoted to finding the most efficient and accurate procedures, protocols, and equipment for screening, diagnosing, and treating children who are deaf or hard of hearing [14]. 

Universal neonatal screening is the most useful procedure for early detection of congenital deafness. Neonatal hearing screening facilitates a confirmed diagnosis of neonatal deafness in the first 4 months of life while without this practice the diagnosis is usually delayed up to 35 m. In the same way, the treatment of children diagnosed from neonatal screening begins before 7 m, and in its absence, it is delayed on average up to 35 m [15,16].

Neonatal hearing screening (<1 month) should check both ears and be universal (>95% of newborns). It must be bilateral because unilateral congenital deafness affects neuropsychological development [7,17,18]. 

**Figure 2:**
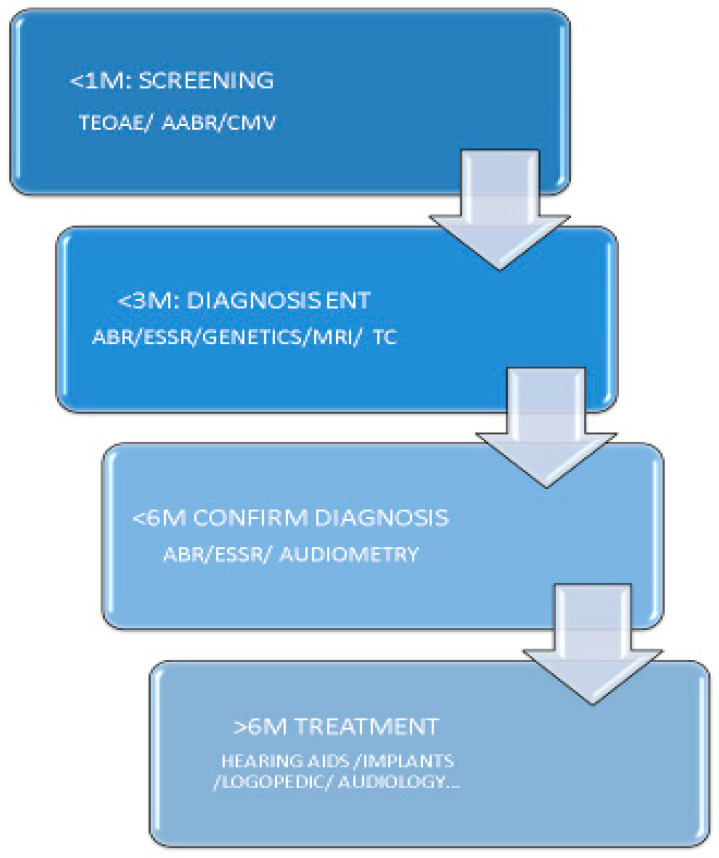
Time goals in hearing screening. Acronyms: TEOAE: transitory evoked otoacoustic emission; AABR: automated auditory brainstem response, CMV: cytomegalovirus; ESSR: steady state potentials; MRI: magnetic resonance, CT: computerized tomography.

Under normal conditions if the technical application is adequate, the referral of patients to otorhinolaryngology (ENT) service will be less than 4%. Diagnostic confirmation must be conducted in good conditions before 3 m and audiologic study before 5–6 m in order to begin treatment at approximately 6 m (Figure 2) [7,18]. 

A universal neonatal screening raises some problems of organization and distribution of tasks related to the responsible hospital service (pediatrics, obstetrics, otorhinolaryngology), health personnel in charge of carrying out the test (pediatrician, nurses, otorhinolaryngologist, etc.), where and when the newborn examination must be performed, and finally, if the study should be carried out in the presence of the parents. In any case, regular check-ups should be made to ensure that all newborns are examined. Likewise, periodic meetings of the services and staff directly involved (obstetrics, pediatrics and otorhinolaryngology) should be scheduled. In any case, the verbal consent of those responsible for the child is required for legal purposes or a written disclaimer kept in neonatal history if family does not want to do the test.

At the present there is controversy about the technical equipment and protocols to be used, but all the programs reduce the age of treatment achieving a better prognosis for hearing and psychosocial adaptation [7,8,9,10]. 

## 3. Types of Neonatal Hearing Screening

When defining the main characteristics of universal neonatal screening for deafness, it must be taken into account that the child will not cooperate and that a large population (all neonates) will be included. Consequently, those procedures should be chosen that, similar to other analytical tests, are sensitive, specific and objective but, given the number of those studied, which are mostly healthy neonates, they must also be atraumatic, simple, repeatable, fast and cheap.

It should be emphasized that screening does not seek a firm diagnosis of the disorder but rather the identification of suspected newborns in order to focus the subsequent effort to its diagnostic confirmation. 

Currently, most neonatal hearing screenings are performed with audiological techniques, mainly those based on acoustic otoemissions (transitory evoked otoacoustic emission, TEOAE) and those based on auditory nerve potentials (automated auditory brainstem response, AABR). Both techniques are sensitive and specific enough to be used in screening but have some differences, the most important of which is the fact that otoemissions only explore hearing up to the cochlear level (which constitutes the most frequent defect with more than 90% cases) but not the neural pathway, which may be affected in a small proportion of cases. On the other hand, otoemissions are faster and cheaper than AABR and require hardly any consumables. They are also less traumatic since they do not need to put electrodes on the skin and are easier to do. Regarding performance, AABR have fewer false positives than otoemissions, especially in the first days after birth [7,8,9,10,19,20,21].

Given that TEOAE do not allow the identification of neural deafness, most authors advise the use of AABR in all children with auditory risk factors due to neural or syndromic pathology [7,8,9,10,18].

Discussion persists about the preferred technique in children without risk factors. The sequential use of both techniques in specific cases (first TEOAE and if they fail, AABR later) is gaining followers, which is facilitated by the manageability of the available devices that can do TEOAE and AABR at the same time [7,8,9,10,22,23].

Currently, more than half of the countries with neonatal hearing screening use TEOAE thanks to its low cost as well as its easy and fast technical implementation, although more programs are increasingly using a mixed protocol (TEOAE-AABR) or, less frequently, only AABR [1,8,9,24,25].

Given the differences in approach on the subject, the Joint Committee on Infant Hearing (JCIH) recommends that each country adopt the most appropriate norms to develop its own protocol in accordance with regional administrative possibilities, government decisions and budget [1]. At the end, it depends on the availability of time and money because AABR costs 50% more than TEOAE and needs double of time although it explores neural pathway and has less false positives. Consequently, there are professionals who always prefer AABR for a more precise diagnosis and fewer losses in the follow-up of infants [7]. 

Concerning the place to carry out the first audiological examination, two alternatives are observed according to local availability of resources, one that conducts the examination at the maternity ward before discharge (assuming more false positives but less losses) and another that prefers to perform the test later in external office (with less false positives and more losses). The former is better performed by all nurses and the latter is better conducted in an external office by dedicated staff that filters the referral to ENT. This is recommended also for all retests in babies that fail the first test [26].

It is necessary to monitor the patient in a longitudinal database, accessible to all specialists involved. It reduces the losses in follow-up that are the Achilles heel of the screening programs!

## 4. Guides and Action Algorithms

**Figure 3:**
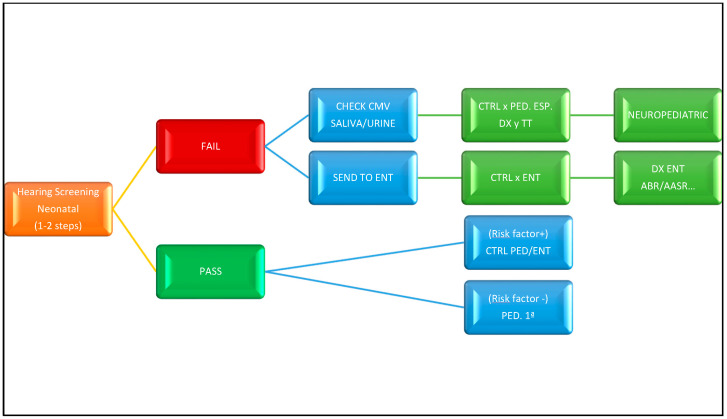
Recommended CODEPEH protocol for newborn hearing screening. Acronyms: CTRL: control; DX: diagnosis, PED: pediatrics, ENT: otorhinolaryngology; CMV: cytomegalovirus.

Figure 3 shows the algorithm of newborn hearing screening recommended by the Spanish “Commission for the Early Detection of Hearing Loss” (CODEPEH) [27].

## 5. Some Details of Practical Interest in Hospital Hearing Screening

There are a series of general and specific aspects that must be taken into account. 

1. The test should be performed on all newborns in a quiet room by trained health personnel that also will fill the database with the results. 

2. Although the study can be carried out by doctors or nurses, either from the pediatric as well as from the otolaryngology or obstetrics services, due to their location and numerical availability, nurses from obstetrics are the most suitable professionals. If possible, it is better that all obstetric nurses are trained in hearing screening because it permits to do the test in all shifts, all day long and covering holidays. Nevertheless, in large hospitals one can dedicate a special team of five health personnel to do it (it improves reliability but worsens continuity because of holidays and leaves).

3. It is advisable to have two devices available in order not to stop testing if one malfunctions. For the same reason, two or three probes, ear tips and cables must be ready.

4. Do not spend more than 5 min on a test. If it is impossible to carry out due to the child’s restlessness or other causes, it must be stopped and repeated later. Try to do it after feeding, in a quieter place or using breastfeeding or a pacifier. 

5. Do not insist on testing, if a test is a “fail” two times, refer to next level after assuring that the ear tip and probe fit, were right and there were no technical problems (too much noise, etc.) [7]. On AABR, check electrical noise and disconnect as much electronic devices as you can (including mobile phones telephones, pulsioximeters, lights, etc.) and prepare well the skin before in order to diminish the impedances (you can use a special gel).

6. In cases where it is required to repeat the test for failing to pass the first test/step it is important to discuss the inpatient versus outpatient retest issue because the former has more failures (because of middle ear status) but a lower lost to follow-ups; the later has opposing figures. The second test/step must be performed by an expert health personnel before 2–3 weeks of age (in order to do a cytomegalovirus test if it fails), it must be bilateral, not testing just the ear that fail before and controlling the time you are spending, if it fails a pair of tries just send it to ENT (Joint Committee on Infant Hearing recommend just one try in good conditions) [7].

7. Parents should be informed as soon as possible of the test results. It is very important to explain what results of the screening test mean: the pass means a normal auditory function but refer means only that technique cannot, at that moment, detect a normal function and that can be also for technical reasons or lack of aeration of middle ear, so another test must be performed in order to confirm the diagnosis some days later. This avoids the excess of anxiety in the family. It is basic to choose the best moment when to communicate results and explain clearly what the screening tests really mean showing the differences of screening versus diagnosis. On the other hand, it is important to remark also the possibility of a late onset hearing loss in spite of a normal screening test. Finally, it is necessary to give all information of this issue in a proper culturally competent way offering an appropriate support [28].

In our experience, keeping these considerations in mind, an effective neonatal hearing screening program can be obtained without too many difficulties. With these tips you can do a sustainable local program.

In any case, no matter how detailed and precise the screening is, the lack of follow-up is frequently observed in those newborns who did not pass the examination in the hospital. There is a high percentage of this babies that do not assist to controls or appointments in external office and also some documentation or contact problems. In most programs represent an 8–10% of the total of babies scheduled for retest [7,9]. According to Centers of Disease Control (CDC), 45% of fails detected have an inappropriate global follow-up. These figures are so important that can run down the results of the whole program. In order to solve this issue, it is necessary to have a good coordination between specialist involved with a responsible of program and a very good database with family address and phone data up to date. In the same line it is very useful to have a person that helps the family to navigate between all tests and appointments to be done. It is also basic to have a good collaboration with primary care pediatricians and other health personnel [29]. The primary care doctors and pediatricians must have a direct telephone number to ask for an appointment in case a baby has been born at home or in another region and has been not screened. In our case, the babies that fail the initial screening, leave the hospital with an appointment to repeat the test before 14 days of life, if they do not return to our external office, we call them for another appointment if possible before 21 days (no more than 1 month). If they do not come, we advise our social services in order to know what the problem is and solve it.

In addition, high risk neonates are controlled in our external office and ENT services in order to detect late onset hearing loss till they have at least two years and usually after that age [18]. 

## 6. Additional Remarks

Some additional problems in neonatal hearing screening deserve to be taken into consideration: infection by cytomegalovirus, the type of lactation and the increasing importance of genetic diagnosis.

Cytomegalovirus infection (CMV) reaches a frequency of about 0.5% of all newborns in developed countries. Most of them are asymptomatic (more than 94%) and about 22% develop either neonatal or late-onset hearing loss.

Approximately 6% are symptomatic and, of these, 33–60% develop hearing loss. The hearing loss is progressive in 11–50% and late-onset in 5–18% of these children. Early diagnosis is important for considering drug treatment with ganciclovir or valganciclovir, given that several studies have shown their usefulness for improving hearing loss or preventing its progression in these children. For a congenital diagnosis it is necessary to have a positive PCR test in urine before 21 postnatal days and that is the reason for doing retest in hearing screening before this date [30,31,32]. 

Breastfeeding is an important factor related to a normal response in TEOAEs test. The reasons are unclear but probably is due to a better middle ear aeration because a more powerful sucking. It may improve the final results of newborn hearing screening reducing the number of neonates who need to be rescheduled for a retest, as well as the associated anxiety and the possibility of losing patients during follow-up. This is another good reason to encourage newborn breastfeeding [33,34].

Finally, it is important to highlight the increasing importance of the genetic component in the origins of congenital deafness; recent research detects genetic abnormalities in up to 80% of cases including more than 400 known syndromes. Of the genetic cases, 70% are non-syndromic and 30% are syndromic. Still, among the non-syndromics, about 80% of the cases hearing loss is related to the autosomal recessive inheritance mechanism and 10–20% to the autosomal dominant one, being that genetic deafness presents all the Mendelian inheritance mechanisms, including the mitochondrial one. 

In the near future the best option for diagnosis could be a newborn hearing screening with the techniques discussed above or new ones (like steady state potentials, etc.), combined with CMV and genetic diagnosis in order to solve the limitations inherent to current neonatal programs because CMV and genetic treatment will be a real option [18,35,36,37,38,39]. 

## 7. Conclusions

Neonatal hearing screening is a necessary, effective and efficient procedure. All techniques currently available are useful, specific and sensitive, although there are other techniques under development (steady state responses, etc.) and also complementary tests (CMV, genetic, etc.) that may improve the current performance of established programs.

It is very important that, if possible, the programs are national in scope and involving primary care, in order to assure measures to control losses in the follow-ups and with post-neonatal audiological protocols for early diagnosis of delayed deafness. Furthermore, and of course, a good communication and implication between the specialists involved is mandatory, ruled by a general coordinator and with a good database to make easy knowing and controlling the quality of the programs, thus justifying the time and money invested.
